# Impact of Alkaline H_2_O_2_ Pretreatment on Methane Generation Potential of Greenhouse Crop Waste under Anaerobic Conditions

**DOI:** 10.3390/molecules23071794

**Published:** 2018-07-20

**Authors:** N. Altınay Perendeci, Sezen Gökgöl, Derin Orhon

**Affiliations:** 1 Environmental Engineering Department, Akdeniz University, 07058 Antalya, Turkey; sezengokgol@hotmail.com; 2 ENVIS Energy and Environmental Systems Research and Development Ltd., ITU ARI Technocity, Maslak, 34469 Istanbul, Turkey; orhon@itu.edu.tr; 3 Environmental Engineering Department, Near East University, Near East Boulevard, 99138 Nicosia/TRNC Mersin 10, Turkey

**Keywords:** alkaline H_2_O_2_ pretreatment, breakdown of lignocellulosic structure, greenhouse crop waste, methane generation, process optimization

## Abstract

This paper intended to explore the effect of alkaline H_2_O_2_ pretreatment on the biodegradability and the methane generation potential of greenhouse crop waste. A multi-variable experimental design was implemented. In this approach, initial solid content (3–7%), reaction time (6–24 h), H_2_O_2_ concentration (1–3%), and reaction temperature (50–100 °C) were varied in different combinations to determine the impact of alkaline H_2_O_2_ pretreatment. The results indicated that the alkaline H_2_O_2_ pretreatment induced a significant increase in the range of 200–800% in chemical oxygen demand (COD) leakage into the soluble phase, and boosted the methane generation potential from 174 mLCH_4_/g of volatile solid (VS) to a much higher bracket of 250–350 mLCH_4_/gVS. Similarly, the lignocellulosic structure of the material was broken down and hydrolyzed by H_2_O_2_ dosing, which increased the rate of volatile matter utilization from 31% to 50–70% depending on selected conditions. Alkaline H_2_O_2_ pretreatment was optimized to determine optimal conditions for the enhancement of methane generation assuming a cost-driven approach. Optimal alkaline H_2_O_2_ pretreatment conditions were found as a reaction temperature of 50 °C, 7% initial solid content, 1% H_2_O_2_ concentration, and a reaction time of six h. Under these conditions, the biochemical methane potential (BMP) test yielded as 309 mLCH_4_/gVS. The enhancement of methane production was calculated as 77.6% compared to raw greenhouse crop wastes.

## 1. Introduction

In the last few decades, there was a drastic change in the conceptual understanding of waste management. Waste is no longer considered as matter to be disposed of at the expense of additional cost, but as a resource. Perhaps the most significant resource component is energy in view of the present and future energy shortages expected, due to demands of rapid population expansion and escalating industrial activities in the world. Therefore, energy recovery from waste is now a hot topic, both in terms of scientific efforts and practical applications.

Recently, renewable energy sources, such as solar energy, wind energy, and geothermal energy, are now being largely explored and exploited. Among these categories, biomass energy should be given specific emphasis mainly due to its accessibility; the energy recovery from biomass is also quite sustainable as the proper disposal of biomass requires costly technical processes. Agricultural waste is an important component of the wide spectrum of waste sources considered within the scope of biomass energy [1]. This study focused on greenhouse agriculture, a significant agricultural practice in areas with a suitable climate, like the Antalya region on the southern coast of Turkey. The Mediterranean region is one of the most important areas in terms of protected cultivation because the mild winter makes production under simple structures possible [2]. Greenhouses provide a protected growing environment that can be controlled during the year. This allows intensive culture with annual yields many times higher than that of field production [3]. Turkey holds an important place in the world for the production of fresh fruit and vegetables, having close to 752,000 decares of greenhouse-covered land, placing it fifth in the world after China, South Korea, Spain, and Japan. About 278,000 decares of greenhouse land is located in the Antalya province, which corresponds to approximately 36.97% of greenhouse land in Turkey. Furthermore, 51% of Turkey’s greenhouse vegetable production (3.2 million tons) is provided by Antalya. Greenhouse agriculture is very significant in the districts of Alanya, Aksu, Elmali, Gazipaşa, Kepez, Korkuteli, Kumluca, Manavgat, and Serik.

While total greenhouse production (tomato, pepper, cucumber, eggplant, and zucchini) was 2,256,325 tons, 1,087,247.75 tons of greenhouse crop waste was produced in the production year of 2005–2006 [4]. Unfortunately, greenhouse cultivation waste lignocellulosic residue is improperly disposed into the environment in Turkey. The conventional disposal methods for most of this waste, such as unconfined storage in forests and road edges, landfilling, and uncontrolled burning, cause significant environmental problems [5]. A limited quantity of greenhouse crop waste is also used for mulching. However, growers prefer not to apply mulching, due to the spread of some diseases and the transfer of non-biodegraded pesticides, herbicides, and others for the subsequent cultivation period. Landfilling is the most applied waste management practice, and results in the release of CH_4_ which is around 20 times more potent as a greenhouse gas (GHG) than CO_2_. Landfilling was shown to be the greatest source of GHG emissions, contributing more than 75% of total emissions associated with waste management [6]. Uncontrolled burning and/or incineration of greenhouse crop waste emits CO_2_ and N_2_O, a GHG gas 310 times more powerful in atmospheric warming than CO_2_. In addition, uncontrolled burning and/or incineration diverts waste from landfill, reducing the amount of methane generated. However, combustion also produces waste in the form of ash. Eventually, waste crops disposed from greenhouses were found to be a renewable and cost-free source of lignocellulosic biomass, whose management is necessary to prevent environmental pollution and to gain an alternative utilization as a fuel biogas. Greenhouse crop waste involves all parts left in the field after the harvest, including roots, stems, leaves, rotten/spoiled vegetables, etc. What makes this category of agricultural waste interesting is its complex lignocellulosic structure, whereby the residue contains cellulose (35–50%), hemicellulose (20–35%), lignin (10–25%), and minor fractions of proteins, oils, and ash [7,8] in such a way that the cellulose is embedded in a lignin–polysaccharide sheet [9]. This structure resists microbial destruction and hydrolysis, and requires pretreatment before an energy recovery process.

Many pretreatment technologies were suggested in the literature, such as physical pretreatment, which generally involves mechanical methods such as shredding and grinding [10,11]. Ultrasonic and microwave methods were also tested [12], but were not recommended due to phenolic by-products and the high energy costs involved [13]. Some physico-chemical methods, based on pretreatment with ammonia [14], hot water, and steam explosion [15,16] were reported, all claiming success; however, they also depend on conditions consuming high energy. Pretreatment conducted under acidic and alkaline conditions [17,18] was also found to be effective in breaking down the lignocellulosic structure.

The delignification process as a means of lignin removal is widely used to bleach high-lignin wood pulps in the pulp and paper industry [19,20]. The application of alkaline H_2_O_2_ is one of the most effective chemical pretreatment approaches for energy recovery from wastes and residues with a lignocellulosic structure. During the alkaline H_2_O_2_ pretreatment, while H_2_O_2_ plays the role of an oxidant, the role of alkaline is to reduce or remove lignin, acetyl, and other uronic substitutions in the hemicellulosic portions of the biomass via swelling, salvation, and saponification, so that the accessibility and digestibility of holocellulose is enhanced [19]. Thus, theH_2_O_2_ delignification of agricultural wastes is strongly pH-dependent, with an optimal pH of 11.5 for the dissociation reaction of H_2_O_2_. During the treatment, alkaline H_2_O_2_ reacts rapidly with lignin to form low-molecular-weight, water-soluble oxidation products. The lignin-oxidizing species is a highly reactive hydroxyl radical (HO·), formed during the degradation of H_2_O_2_ in a reaction with the hydroperoxy anion (HCOO^−^). HCOO^−^ is the active species and is responsible for the bleaching action of H_2_O_2_ under alkaline conditions. On the other hand, hydroperoxyl and hydroxyl radicals generated by the decomposition of H_2_O_2_ are responsible for solubilizing hemicelluloses [21]. This process also has the advantage of not leaving H_2_O_2_ residue, and it is considered as an environmentally friendly and low-cost application [22]. While a large number of studies were conducted using alkaline H_2_O_2_ pretreatment on various types of agricultural waste, such as corn stover, wood waste, soft wood, cashew apple bagasse, energy crops, sugar cane bagasse, agricultural crop stalks, and cotton stalks [19,22,23,24,25,26,27,28,29], this method, although quite promising, remains untested for greenhouse crop wastes.

In this context, the main objective of the study was to carry out an experimental assessment of the effect of alkaline H_2_O_2_ pretreatment on the biodegradability and the methane generation potential of greenhouse crop wastes. A central composite design (CCD) of response surface methodology (RSM) was applied to determine the optimal process conditions of alkaline H_2_O_2_ pretreatment for maximum biogas production in the most cost-effective way. H_2_O_2_ concentration, initial solid content, reaction temperature, and reaction time were selected as independent variables. The effects of these four independent variables on soluble chemical oxygen demand (COD), soluble reducing sugar, total lignin on an extractives free bases, and methane generation potential were investigated in detail. The alkaline H_2_O_2_ pretreatment process was optimized to enhance methane production assuming a cost-driven approach. The effects of the alkaline H_2_O_2_ pretreatment process on the molecular-bond characterization and surface properties of greenhouse crop waste were also examined via Fourier-transform infrared spectroscopy (FTIR) and scanning electron microscopy (SEM). To the best of our knowledge, this is the first study on biogas production from greenhouse crop waste with the integration of an alkaline H_2_O_2_ pretreatment process. 

## 2. Results and Discussion

### 2.1. Chemical Composition

The greenhouse crop waste used in the experiment contained around 13.6% dry matter, indicating an average moisture content of more than 86%. The organic fraction of the dry solids, i.e., volatile solids (VS), was measured as 68.7%, mostly composed of lignocellulosic material. The characteristics of the greenhouse crop waste, expressed in terms of major parameters, are presented in Table 1. The cellulose, hemicellulose, lignin, and soluble matter contents of the fresh greenhouse crop waste were measured as 19.49%, 3.89%, 0.03%, and 76.58%, respectively. The elemental composition of the fresh greenhouse crop waste was found to be 29.23% C, 4.89% H, and 2.96% N. The general composition profile reflected in the Table 1 is different from a previous assessment of the same waste [5], which had a different composition. While the composition of mixed greenhouse crop waste was 61.71% tomato, 22.44% cucumber, 7.92 % eggplant, 5.72 % pepper, and 2.21% zucchini in the previous work [5], the composition in this work was 72% tomato, 14.31% cucumber, 5.11% eggplant, 6.69% pepper, and 1.88% zucchini. Furthermore, the green house crop waste used in the previous study [5] was obtained from the Kumluca region, located in west Antalya. On the other hand, the green house crop waste in this study was acquired from the Gazipaşa region, located in east Antalya. Conclusively, even though the sampling period was the same, the location and composition of the collected greenhouse crop waste was different. Specifically, the cellulose and hemicellulose contents, together with the carbon content, were found to be lower. The reason is most likely due to sampling done from different cultivation areas, with a different sample composition. 

The total COD equivalent of the organic matter in the crop waste was determined as 1.49 gCOD/gVS. This is a significant stoichiometric ratio, quite similar to the *f_X_* value of 1.4 gCOD/gVS, characteristic of biomass in activated sludge systems. This ratio corresponds to the traditional empirical formula of C_5_H_7_NO_2_, which is still in use for the basic stoichiometry of activated sludge [30]. While noting that the measured nitrogen content remains somewhat lower, it would be acceptable to adopt this simplified formula for the COD–VS relationship in greenhouse crop waste.

Table 1 also indicates the magnitude of COD leakage into the solution (soluble COD (sCOD), *S_T_*) as 61 mgCOD/gVS, and the soluble reducing sugar (sRedSugar) content in this leakage as 7.6 mgCOD/gVS. It should be noted that the soluble sugar component is basically the same as the readily biodegradable COD fraction (*S_S_*) identified in wastewater [31,32]. It is interesting to note that Sözen et al. [33] reported 5250 mg of COD leakage from 90 g of domestic sludge, quite similar to the 58 mg of *S_T_* per g of dry sludge in “eluate tests” performed for evaluating compliance with the limitation of dissolved organic carbon for the landfilling of municipal treatment sludge.

### 2.2. Effect of Alkaline H_2_O_2_ Pretreatment

**Figure 1:**
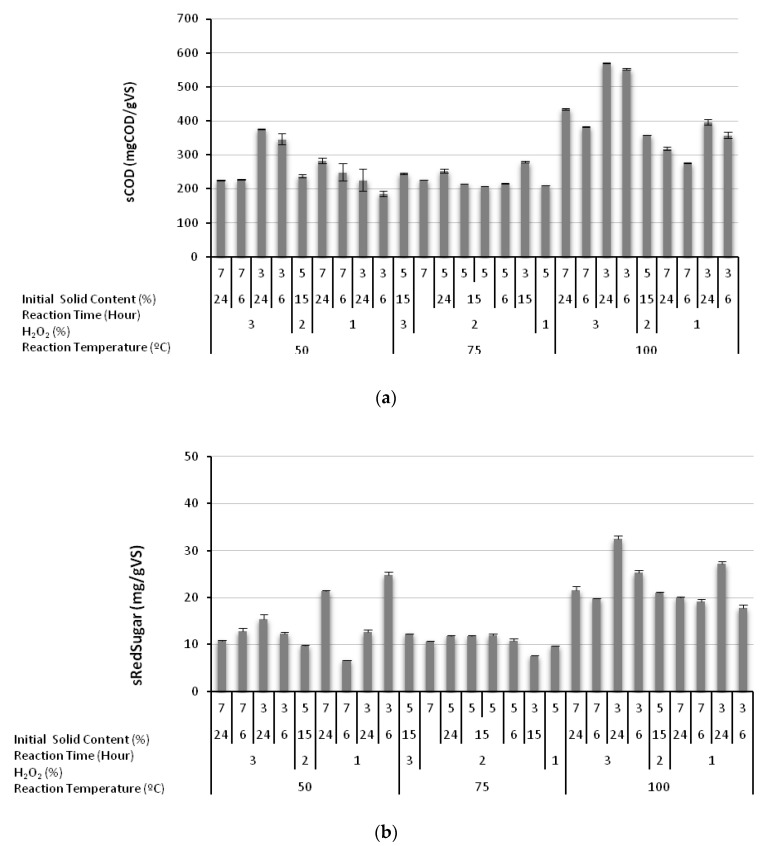
Increase in soluble chemical oxygen demand (sCOD; (**a**)) and increase in soluble reducing sugar (sRedSugar; (**b)**) due to alkaline H_2_O_2_ treatment.

The directly observable effect of alkaline H_2_O_2_ treatment was the substantial increase in the magnitude of sCOD, as illustrated in Figure 1a. All values in Figure 1a were compared with the sCOD value of 61 mgCOD/gVS in the original raw greenhouse crop waste, in order to visualize the effect of alkaline H_2_O_2_ treatment. Basically, Figure 1a shows that (i) sCOD (*S_T_*) was increased above 200 mgCOD/gVS in all tests; (ii) the most noticeable increase was observed in experiments conducted at 100 °C; in a few experimental runs, *S_T_* exceeded 500 mgCOD/gSV, corresponding to more than an 800% increase compared with the initial COD leakage capacity of the greenhouse crop waste; (iii) the sCOD increase always remained higher when the reaction time was raised to 24 h while other parameters remained the same. This observation is particularly important, since it shows that the H_2_O_2_ dosage was adjusted to increase the amount of sCOD, but not to oxidize and chemically remove the sCOD generated.

Figure 1b shows that alkaline H_2_O_2_ treatment also increased the soluble sugar (sRedSugar) leakage. The highest sRedSugar concentration was found to be 32.47 mg of glucose/gVS from the greenhouse crop waste pretreated at a reaction temperature of 100 °C, an H_2_O_2_ concentration of 3%, a reaction time of 24 h, and 3% initial solid content, which are the same pretreatment conditions where the maximum increase in sCOD was observed (Figure 1a). It should be remembered that the sRedSugar/sCOD ratio of the greenhouse crop waste before treatment was 12.4% (Table 1). The values displayed in Figure 1b indicate that, while sRedSugar values also increased with H_2_O_2_ treatment, the sRedSugar/sCOD ratio decreased from 12.3% to in the range of 3.9–7.8%.

**Figure 2:**
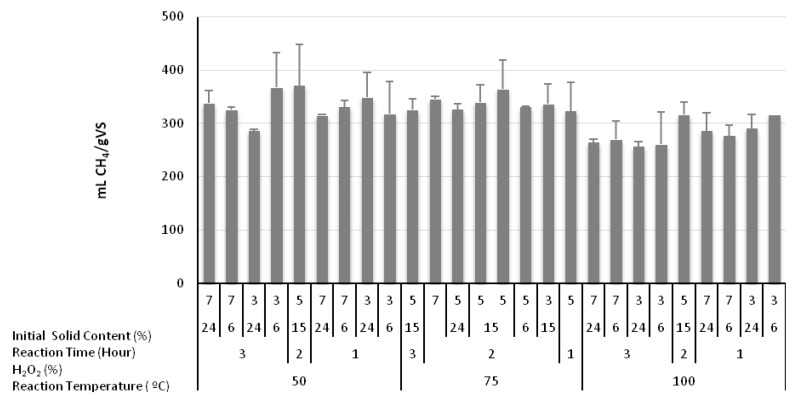
Methane generation due to the impact of alkaline H_2_O_2_ pretreatment.

The effect of alkaline H_2_O_2_ treatment could only be quantified and evaluated in comparison with the methane generation of the raw greenhouse crop waste without pretreatment. The volume of methane produced from the raw greenhouse crop waste was 174 mLCH_4_/gVS. The experimental outcomes for the biochemical methane potential (BMP) test from the pretreatment experiments are presented in Figure 2. After pretreatment, the highest BMP value was 370.9 mLCH_4_/gVS, obtained at a reaction temperature of 50 °C, an H_2_O_2_ concentration of 2%; a reaction time of 15 h, and 5% initial solid content, while the the lowest BMP value (256.6 mLCH_4_/gVS) was obtained from the greenhouse crop waste pretreated at a reaction temperature of 100 °C, an H_2_O_2_ concentration of 3%, a reaction time of 24 h, and 3% initial solid content. It can be concluded that the dependent variables of sCOD and sRedSugar, which had the maximum values under these conditions. behaved differently than the variable of BMP.

It should be remembered that an initial sCOD amount of 61 mg/gVS was also measured in the greenhouse crop waste. Based on the ratio of 0.35 LCH_4_/gCOD, now universally recognized as the relationship between sludge COD utilized and methane generated [34], the utilization of the available sCOD would only correspond to 21 mLCH_4_/gVS. The generation of the remaining 153 mLCH_4_/gVS has to be related to the hydrolysis of the particulate organics, requiring 0.437 g of particulate COD/gVS. This particulate COD consumption may be converted to 0.31 gVS/gVS, using the previously selected ratio of 1.4 gCOD/gVS. In short, biochemical reactions for raw greenhouse crop waste depleted all available sCOD, and broke down/hydrolyzed 31% of the existing volatile solids, converting them into methane.

The increase in magnitude of methane generation was obviously a direct observation of the effect of alkaline H_2_O_2_ treatment. The first important observation is the escalation in the volume of collected methane to a narrow bracket of 250–350 mLCH_4_/gVS as a result of alkaline H_2_O_2_ treatment. The second is the relatively lower methane volumes of around 250 mLCH_4_/gVS associated with the experimental runs conducted at 100 °C, despite much higher sCOD levels achieved in the same experiments.

**Figure 3:**
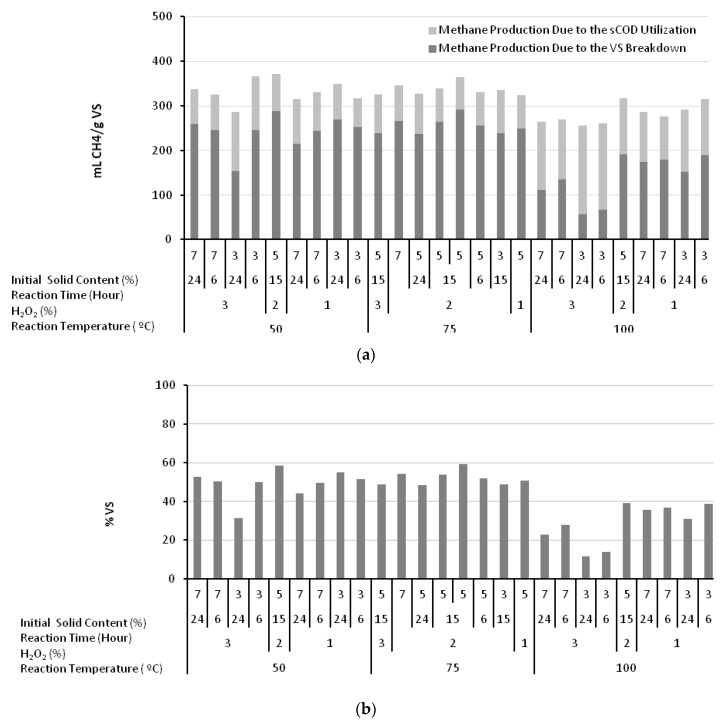
Methane production from sCOD and volatile solid (VS) destruction (**a**) and VS breakdown (**b**) due to the impact of alkaline H_2_O_2_ treatment.

This effect may be further evaluated in terms of (i) the increase in the sCOD levels, and (ii) changes in the levels of particulate organic matter hydrolysis for this purpose. The related evaluations are plotted in Figure 3a,b, which show both the relative contributions of sCOD and the particulate matter hydrolysis. From a different perspective, in the experimental conditions describing a reaction temperature of 50 °C, an H_2_O_2_ concentration of 3%, 7% initial solid content, and a reaction time of 24 h, only 78.8 mLCH_4_/gVS was related to the available sCOD, while 258.8 mLCH_4_/gVS was produced from the hydrolysis of 52.8% VS. Whereas at a reaction temperature of 100 °C, an H_2_O_2_ concentration of 3%, 3% initial solid content, and a reaction time of 24 h, the increased amount of sCOD produced 199.7 mL of the 256.6 mLCH_4_/gVS generated, while particulate organic matter hydrolysis remained limited to 11.6%. On this basis, the role of the particulate COD breakdown and hydrolysis seemed reversed at high temperatures. The limitation of methane generation under these conditions may be attributed to the formation of inhibitory by-products likely to be formed during H_2_O_2_ oxidation.

**Figure 4:**
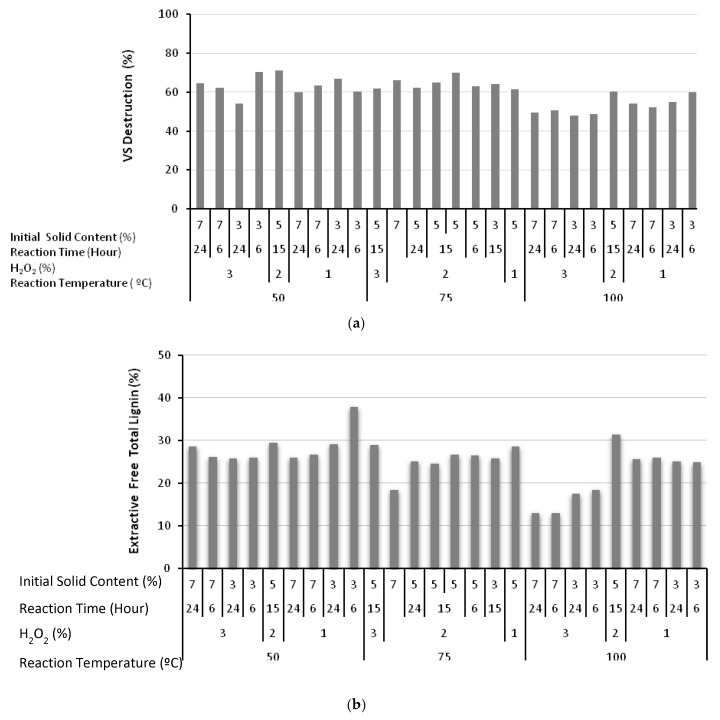
Destruction profile for volatile solids due to the impact of alkaline H_2_O_2_ treatment (**a**). Experimental outcomes for the total lignin on an extractives free bases (**b**).

The utilization rate of particulate organic matter under anaerobic conditions is an important parameter that reflects the biodegradability characteristics of the waste. The chemical structure of the greenhouse crop waste, dominated by lignocellulosic material, is too complex for biodegradation under natural conditions. In fact, the experiments indicated that only 31% of the waste could be utilized to generate methane without any pretreatment. Alkaline H_2_O_2_ treatment breaks down this complex chemical structure and hydrolyzes it into simple/soluble compounds, detectable by the increase in the magnitude of sCOD. This process significantly affects and increases the biodegradation of the waste. The destruction of the volatile solids takes place in two steps: (i) initial conversion into sCOD, and (ii) partial utilization of volatile solids under anaerobic conditions. For example, at a reaction temperature of 50 °C, an H_2_O_2_ concentration of 3%, 7% initial solid content, and a reaction time of 24 h, the incremental sCOD increase between the pretreated and raw samples (ΔsCOD) was 164.4 mgsCOD/gVS, corresponding to a VS hydrolysis (ΔVS) of 0.117 gVS/gVS. The generation of 338 mLCH_4_/gVS additionally consumed 0.528 gVS/gVS, with an overall VS destruction calculated as 64.6%. Furthermore, at a reaction temperature of 100 °C, an H_2_O_2_ concentration of 3%, 3% initial solid content, and a reaction time of 24 h, ΔsCOD was measured as 509.8 mg/gVS, representing an initial VS hydrolysis of 0.364 gVS/gVS. An additional amount of volatile solids (ΔVS) of 0.116 gVS/gVS was also converted into methane, resulting in a lower VS destruction of 48%. These values should be compared with the 40–50% volatile matter utilization in the anaerobic digestion of sewage sludge [35]. The VS utilization profile achieved with alkaline H_2_O_2_ treatment is plotted in Figure 4a. The decrease in utilization rate at high sCOD levels also confirmed the presence and effect of inhibitory oxidation by-products. Furthermore, the experimental outcomes for the total lignin on an extractives free bases are presented in Figure 4b. As plotted in Figure 4b, the lowest total lignin on an extractives free bases was measured as 13.1% from the greenhouse crop waste pretreated at a reaction temperature of 100 °C, an H_2_O_2_ concentration of 3%, a reaction time of 24 h, and 7% initial solid content. It should be remembered that the second lowest BMP value of 264.2 mLCH_4_/gVS was also observed under these conditions.

### 2.3. Alkaline H_2_O_2_ Pretreatment Process Optimization

The accuracy of the models was explained by the determination coefficient (*R*^2^) and coefficient of adjusted determination (Adj-*R*^2^). The *R*^2^ values were found to be 0.9682, 0.7740, 0.8376, and 0.5728 for the sCOD, sRedSugar, total lignin on an extractives free bases, and BMP, respectively, whereas the Adj-*R*^2^ values were calculated as 0.9562, 0.6966, 0.7762, and 0.4112. The *R*^2^ and Adj-*R*^2^ values for the models of sCOD, sRedSugar, and total lignin on an extractives free bases in Table 2 indicated that acceptable fits were obtained between the response and the independent variables. However, only moderate *R*^2^ and Adj-*R*^2^ values were calculated for the BMP model. Quadratic regression models were strongly considerable, as it was apparent from Fisher’s *F*-test with very low probability outcomes (*p*-value *>* F = 0.0001 for sCOD, sRedSugar, total lignin on an extractives free bases, and BMP).

Since the objective of alkaline H_2_O_2_ pretreatment was the enhancement of methane production with a reasonable process cost, process optimization of alkaline H_2_O_2_ pretreatment was executed based on minimizing the cost of the process (cost-driven approach) using the models developed for sCOD, sRedSugar, total lignin on an extractives free bases, and BMP. In the cost-driven optimization approach, the dependent variables of sCOD and total lignin on an extractives free bases were set in range, whereas sRedSugar (+) and BMP (+) were maximized. On the other hand, the independent variables of reaction temperature (+++++), reaction time (+++++), and H_2_O_2_ concentration (+++++) were minimized, while VS content (+++++) was maximized.

Optimal alkaline H_2_O_2_ pretreatment conditions were determined with the highest desirability of 0.917 at a reaction temperature of 50 °C, 7% initial solid content, an H_2_O_2_ concentration of 1%, and a reaction time of six h under these restraints. The optimal values for sCOD, sRedSugar, total lignin on an extractives free bases, and BMP were predicted to be 296.4 mgsCOD/gVS, 102.1 mg sRedSugar/gVS, 28.7%, and 318.6 mLCH_4_/gVS, respectively, using the models. An alkaline H_2_O_2_ pretreatment experiment using a cost-driven approach conditions was performed for validation of the process optimization. The values of sCOD, sRedSugar, total lignin on an extractives free bases, and BMP were measured as 290.3 mgsCOD/gVS, 106.9 mg sRedSugar/gVS, 28.1%, and 309 mLCH_4_/gVS, respectively, supporting the predictive power of the developed models. The BMP enhancement was calculated as 77.6% compared to the raw greenhouse crop waste under the conditions optimized for the process cost.

**Figure 5:**
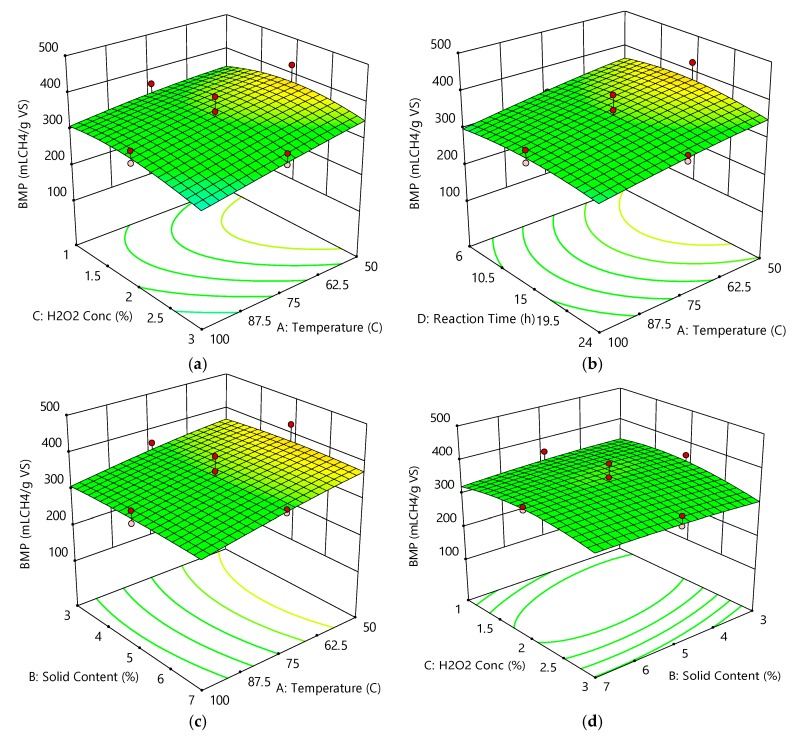

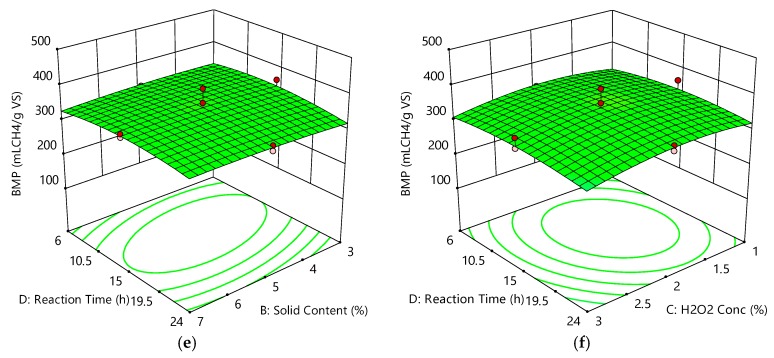
Effects of independent variables on biochemical methane potential (BMP). (**a**) H_2_O_2_ concentration and temperature; (**b**) reaction time and temperature; (**c**) solid content and temperature; (**d**) H_2_O_2_ concentration and solid content; (**e**) reaction time and solid content; (**f**) reaction time and H_2_O_2_ concentration.

Three-dimensional (3D) graphs were employed to emphasize the impacts of independent variables under optimal conditions. The effects of independent variables on BMP are demonstrated in Figure 5a–f. In Figure 5a, BMP decreased due to increasing H_2_O_2_ concentration at a reaction temperature of 100 °C, whereas BMP increased due to decreasing reaction temperature (from 100 °C to 50 °C) within the range of 1–3% H_2_O_2_ concentration. A maximum predicted BMP enhancement of 106.9% compared to the raw greenhouse crop waste was observed at a reaction temperature of 68 °C and an H_2_O_2_ concentration of 2%. In Figure 5b, c, BMP decreased when the reaction temperature was increased to 100 °C at a reaction time of 24 h and 7% initial solid content. When the reaction time was maintained at 24 h, a decrease in BMP was observed when the temperature was increased to 100 °C. Similarly, when the initial solid content was kept constant at 7%, the decrease in BMP was temperature has a negative impact on BMP. Furthermore, as seen in Figure 5d–f, BMP was not affected by the interactive effects of H_2_O_2_ concentration with initial solid content, reaction time with initial solid content, and reaction time with H_2_O_2_ concentration. A maximum BMP was obtained at 4–6% initial solid content, H_2_O_2_ concentrations of 1.5–2.5%, and reaction times of 10–18 h.

### 2.4. Chemical Structure and Morphological Changes of Biomass

**Figure 6:**
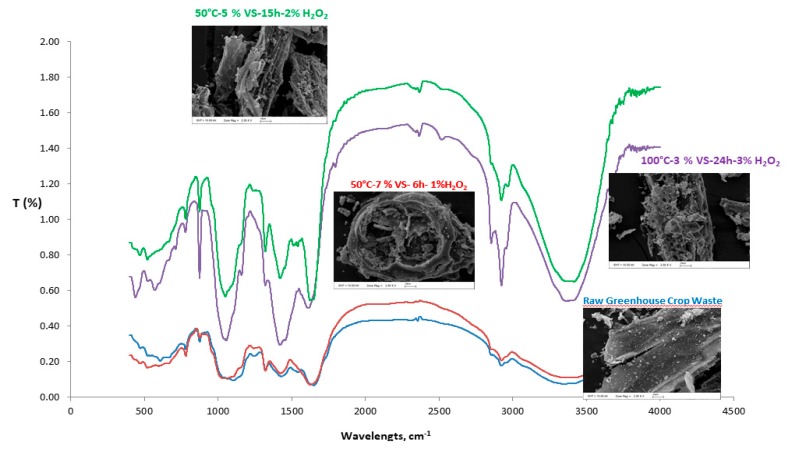
Fourier-transform infrared (FTIR) spectra and SEM images of raw and pretreated greenhouse crop waste.

The FTIR spectra and SEM images of greenhouse crop waste pretreated with alkaline H_2_O_2_ under different conditions (50 °C, 5% VS, 15 h, 2% H_2_O_2_ for maximum CH_4_ production; 100 °C, 3% VS, 24 h, 3% H_2_O_2_ for maximum sCOD and sRedSugar production, along with minimum CH_4_ production; and 50 °C, 7% VS, 6 h, 1% H_2_O_2_ for cost optimization) compared to those of the raw greenhouse crop waste are presented in Table 3 and Figure 6.

As seen in Figure 6, the spectral profiles and relative intensities of the bands belonging to the raw greenhouse crop waste and that pretreated with alkaline H_2_O_2_ were found to be very similar under conditions of 50 °C, 7% VS, 6 h, and 1%H_2_O_2_ for cost optimization. On the other hand, the spectral profiles were different from the raw greenhouse crop waste for that pretreated with alkaline H_2_O_2_ under conditions of 50 °C, 5% VS, 15 h, and 2% H_2_O_2_ for maximum CH_4_ production, and that pretreated with alkaline H_2_O_2_ under conditions of 100 °C, 3% VS, 24 h, and 3% H_2_O_2_ for maximum sCOD and sRedSugar production, along with minimum CH_4_ production. New peaks were observed after alkaline H_2_O_2_ pretreatment, indicating that the chemical composition of greenhouse crop waste changed. In particular, the prominent absorbances at 895–900, 1050, 1270, 1430–1460, 1510–1600, 2920–2925, 3420, and 3446 cm^−1^ in the spectra were relatively different from the spectrum of raw greenhouse crop waste. As clearly seen in Table 3, the lignin-related absorbance values observed at 1270, 1430–1460, and 1510–1600 cm^−1^ revealed that the alkaline H_2_O_2_ pretreatment was effective on lignin disintegration. Sun et al. [19] also stated that the delignification of agricultural crop stalks could occur during the alkaline H_2_O_2_ pretreatment process, while the macromolecular structure of cellulose did not show any noticeable change. Results from this study confirm the findings of Sun et al. [19].

As seen in Figure 6, the raw greenhouse crop waste exhibited a smooth, non-porous, compact, and rigid surface structure. There was no separation of fibers, or ruptures and scars. On the other hand, the pretreated greenhouse crop waste demonstrated a rough and porous structure. In particular, the fibrils of greenhouse crop waste pretreated with alkaline H_2_O_2_ under conditions of 100 °C, 3% VS, 24 h, and 3% H_2_O_2_ were completely deformed, and their structural integrity was disrupted. The SEM examination revealed that the morphological changes, along with the tissue damage, resulted from the alkaline H_2_O_2_ pretreatment. Similar to our findings, Rezende et al. [36] also stated that alkaline and NaCl pretreatment dissolved the inter-fibrillar or bulk lignin, while disrupting the initial fiber structure, leading to the disaggregation of micro-fibrils from their neighboring fibers.

## 3. Materials and Methods

### 3.1. Experimental Rationale

Antalya is the largest area for greenhouse cultivation in Turkey, providing tomato, pepper, cucumber, eggplant, and zucchini. Greenhouse crop waste, consisting of roots, stalks, leaves, and fruits from cultivation, is generated in the region, creating environmental problems. The greenhouse crop waste was supplied by the growers, and fresh waste was sliced into approximately 1cm pieces, and was stored in sealed plastic bags at −20 °C until used for composition analyses, alkaline H_2_O_2_ pretreatment experiments, and methane generation potential tests.

The first phase of the experiments involved the characterization of the greenhouse crop waste in terms of the parameters that would be used as major indicators for the extent of energy recovery achieved by means of alkaline H_2_O_2_ treatment.

Analyses of the total solids (TS) and volatile solids (VS) were performed based on standard methods 2540C [40]. Analyses of the total chemical oxygen demand (COD) were done according to standard methods 5220B [40]. The Kjeldahl nitrogen was determined using a Kjeldahl nitrogen analyzer (Büchi Digest Automat K-438, Büchi Auto Kjeldahl Unit K-370 and Radiometer TitraLab 840, Büchi, Flawil, Switzerland). The contents of lignin, cellulose, hemicellulose, and soluble matter were determined according to the Van Soest procedure [41] using a Gerhard FBS6 (Gerhard, Königswinter, Germany). Analyses of the total free lignin of extractives (acid-insoluble and acid-soluble) were performed according to the “Determination of Structural Carbohydrates and and Lignin in Biomass, NREL/TP-510-42618” [42]. The protein concentration was determined using the Lowry method [43]. The extractive matter and lipid contents of samples were determined using Soxhlet extraction [44]. The soluble chemical oxygen demand (sCOD) was determined using a Hach-Lange DR5000 spectrophotometer (Hach Lange GmbH, Duesseldorf, Germany) and a Lange LT200 (Grasscht, Germany) with COD kits. The concentrations of soluble reducing sugar (sRedSugar) were determined via the Dinitrosalicylic acid (DNS) method [45]. The elemental composition of the greenhouse crop waste was identified using a CHNS elemental analyzer (LECO, CHNS-932, St. Joseph, MI, USA). All composition analyses were executed in triplicate, and the quotable outcomes are demonstrated as means.

### 3.2. Alkaline H_2_O_2_ Pretreatment Experiments

The greenhouse crop waste was pretreated in a Parr reactor (Parr Instrument Company) with a 200 mL working volume. The independent variables with a potential impact on alkaline H_2_O_2_ pretreatment were selected as reaction temperature (50–100 °C), H_2_O_2_ concentration (1–3%), reaction time (6–24 h), and initial solid content of greenhouse crop waste (3–7%). The pretreatment experiments were done in duplicate under each condition. The calculated amount of fresh greenhouse crop waste and H_2_O_2_ solution (*w*/*w*) was loaded into the pretreatment reactor, and initial pH values were set to 11.5 using 6M NaOH solution, with the reactors heated to the appropriate reaction temperature. When the predetermined temperature was attained, the experiment time was started. After reaching the determined reaction time, the reactor was put into ice and a water bath to cool down and stop the reaction. The pretreatment process was evaluated according to sCOD, sRedSugar, total free lignin of extractives, and BMP as objective functions related to pretreatment yield. The samples were centrifuged at 15,000 rpm for 10 min for the sCOD and sRedSugar analyses. The amount of sCOD was determined using a Hach-Lange DR5000 spectrophotometer and a Lange LT200 (Grasshut, Germany) with COD kits. The sRedSugar concentrations were determined via the DNS method [45]. Analyses of the total free lignin of extractives (acid-insoluble and acid-soluble) were performed according to the “Determination of Structural Carbohydrates and and Lignin in Biomass, NREL/TP-510-42618” [42] using the solid phase of the pretreated samples. The remaining pretreated samples containing solid and liquid fractions were stored at −20 °C for the subsequent methane generation potential experiment.

### 3.3. Methane Generation Potential Experiment

The efficiency of alkaline H_2_O_2_ pretreatment was determined using a biochemical methane potential test (BMP) based on methane production. The samples, including macro and micro nutrients, were incubated in a closed glass reactor with a specific quantity of seed sludge (inoculum). Mesophilic conditions (35 °C) were preferred for the BMP tests. The BMP protocol according to Carrère et al. and Us & Perendeci [5,46] was implemented. For the BMP tests, 500 mL glass reactors with a working volume of 400 mL were filled with sample, seed sludge, nutrients, and a tampon solution. All BMP reactors were loaded with seed sludge from the anaerobic reactor of an Antalya city wastewater treatment plant. Fifty-six glass reactors were used in the study, and two of them were fed with only seed sludge and nutrients to specify the methane potential of seed sludge on its own. The 52 glass reactors were used with different pretreated samples, and two reactors containing raw greenhouse crop waste were used as controls. After the optimization of conditions for alkaline H_2_O_2_ pretreatment, the BMP test was also conducted under optimal conditions with two duplicates for validation of the model. The food-to-microorganism ratio (F/M) was fixed at 0.5 (gVS waste/gVS inoculum) for the glass reactors. The initial pH was set to neutral for all reactors. To keep anaerobic conditions in the reactors, a gas mixture of N_2_/CO_2_ (70/30%) was flushed. The BMP test lasted for 62 days. The produced biogas was measured based on a gas-water displacement method. The biogas composition was ascertained using gas chromatography (GC; Varian 4900). A standard gas consisting of 60% (*v*/*v*) CH_4_ and 40% CO_2_ was used for the calibration of gas chromatography. The gas production of seed sludge was counted in the computation of biogas production of the samples. The methane production was estimated as mL of methane per g of VS (mLCH_4_/gVS) added to the reactor.

### 3.4. Optimization of the Alkaline H_2_O_2_ Pretreatment Process

The pretreatment process was optimized using a CCD of RSM. Three levels of four independent variables were applied for the CCD, using the Design-Expert^®^ software (Minneapolis, MN, USA). The ranges of each independent variable were established based on information in the literature and on our previous experimental experience. The levels of the independent variables were coded as −1 and +1. The four independent variables were changed within the following ranges: 50–100 °C (reaction temperature), 6–24 h (reaction time), 1–3% (H_2_O_2_ concentration), and 3–7% (initial solid content). A total of 52 runs, including four runs at the design center and duplicates of each run, were determined using a CCD.

The performance of the alkaline H_2_O_2_ pretreatment process was evaluated based on sCOD, sRedSugar, total free lignin of extractives, and the BMP test as dependent variables. The outcomes from the pretreatment experiments were modeled using the Design-Expert^®^ software (Minneapolis, MN, USA). Analyses of the regression coefficients, variance (ANOVA), and the *p*- and *F*-values were preferred for the model assessment. The adequacy of the model fit was presented by the coefficient of determination (*R*^2^) and the adjusted determination coefficient (Adj-*R*^2^).

The alkaline H_2_O_2_ pretreatment process was also optimized using the optimization module of the Design-Expert^®^ software (Minneapolis, MN, USA). The optimization of the alkaline H_2_O_2_ pretreatment process was executed using the models developed for sCOD, sRedSugar, total free lignin of extractives, and BMP. The goal settings were carried out using the plus (+) symbols in the Design-Expert^®^ program (Minneapolis, MN, USA).

### 3.5. Fourier-Transform Infrared (FTIR) Spectroscopy and Scanning Electron Microscopy (SEM)

Changes in the molecular-bond characterization of greenhouse crop waste were evaluated using an ATR-FTIR-Varian 1000 model FTIR spectrometer. The measurements were analyzed by averaging the signal of 16 scans across the range of 500 cm^−1^ to 4000 cm^−1^ with a spectral resolution of 4 cm^−1^. The evaluation of deformations on the surface of the greenhouse crop waste was also investigated, using a Zeiss Leo 1430 scanning electron microscope at a voltage of 15 kV.

## 4. Conclusions

In the light of the experimental results and evaluations reported in the preceding sections, a number of concluding remarks could be drawn for this study.

The alkaline H_2_O_2_ pretreatment partially destroyed the complex lignocellulosic structure of the greenhouse crop waste. The organic matter was initially broken down and then hydrolyzed into simple, soluble compounds. On this basis, the alkaline H_2_O_2_ pretreatment induced a significant increase in the range of 200–800% in COD leakage into the soluble phase, and boosted the methane generation potential from 174 mLCH_4_/gVS to a much higher bracket of 250–350 mLCH_4_/gVS. Similarly, the volatile matter utilization increased from 31% in the waste material before treatment to 50–70% after treatment, depending on the selected experimental conditions.

The alkaline H_2_O_2_ pretreatment was optimized to determine the optimal conditions for the enhancement of methane generation assuming a cost-driven approach. The optimal alkaline H_2_O_2_ pretreatment conditions were found to be a reaction temperature of 50 °C, 7% initial solid content, an H_2_O_2_ concentration of 1%, and a reaction time of six h. Under these conditions, the BMP test yielded a production of 309 mLCH_4_/gVS. The enhancement of methane production was calculated as 77.6% compared to raw greenhouse crop waste.

The results obtained provide an optimistic perspective for the possibility of energy recovery from complex waste such as greenhouse crop waste. It is recommended that future studies be directed toward testing new pretreatment processes, as well as toward novel energy recovery technologies such as pyrolysis, instead of traditional anaerobic digestion.

## Figures and Tables

**Table 1:** Average characteristics of the greenhouse crop waste.

Parameter	Result
Total solid, TS (g/kg)	136.53
Volatile solid, VS (g/kg)	93.9
Total Kjeldahl nitrogen, TKN (mg/gVS)	6.75
Protein (mg/gVS)	60
Chemical oxygen demand, COD (mg/gVS)	1494.1
Soluble chemical oxygen demand, sCOD (mg/gVS)	60.88
Soluble reducing sugar, sRedSugar (mg/gVS)	7.59
Extractable material and lipids * (%)	0.14
Van Soest fractionation	
Soluble matter (%)	76.58
Hemicellulose (%)	3.89
Cellulose (%)	19.49
Lignin (%)	0.03
Total lignin on an extractive free bases (%)	19.39
Acid-insoluble (%)	17.25
Acid-soluble (%)	2.11
**Elemental Analysis**	
C (%)	29.23
H (%)	4.89
N (%)	2.96
S (%)	1.1

* Determined in extractives soluble in water.

**Table 2:** ANOVA results for sCOD, sRedSugar, total lignin on an extractives free bases, and biochemical methane potential (BMP) models.

**sCOD Model**			
Quadratic model			
Prob > F	<0.0001 Significant	Adj-*R*^2^	0.9562
*R*^2^	0.9682	Pred-*R*^2^	0.9338
Adeq Precision	35.6593	C.V%	8.85
sCOD = +1045.11218 − 24.76191 × Reaction temp. − 44.99164 × Solid content + 88.00049 × H_2_O_2_ concent. − 3.98184 × Reaction time − 0.64327 × Reaction temp. × Solid content + 1.48441 × Reaction temp. × H_2_O_2_ concent. + 0.022507 × Reaction temp. × Reaction time − 27.45672 × Solid content × H_2_O_2_ concent. − 0.000607639 × Solid content × Reaction time − 0.62899 × H_2_O_2_ concent. × Reaction time + 0.19592 × Reaction Temp.^2^ + 11.76235 × Solid content^2^ + 6.39440 × H_2_O_2_ concent.^2^ + 0.21604 × Reaction time^2^.			
**sRedSugar Model**			
Quadratic model			
Prob > F	<0.0001 Significant	Adj-*R*^2^	0.6966
*R*^2^	0.7740	Pred-*R*^2^	0.5519
Adeq Precision	11.705	C.V%	41.85
sRedSugar = +844.41473 − 18.34946 × Reaction temp. + 16.89274 × Solid content − 136.48065 × H_2_O_2_ concent. – 13.05242 × Reaction time − 0.17577 × Reaction temp. × Solid content + 1.01831 × Reaction temp. × H_2_O_2_ concent. + 0.063115 × Reaction temp. × Reaction time − 2.93797 × Solid content × H_2_O_2_ concent. + 0.27415 × Solid content × Reaction time + 0.12308 × Reaction temp.^2^ − 1.32058 × Solid content^2^ + 18.98017 × H_2_O_2_ concent.^2^ + 0.29090 × Reaction time^2^			
**Total Lignin on an Extractives Free Bases Model**			
Quadratic model			
Prob > F	<0.0001 Significant	Adj-*R*^2^	0.7762
*R*^2^	0.8376	Pred-*R*^2^	0.6727
Adeq Precision	14.903	C.V%	14.18
1/(Lignin) = +0.0736566 + 5.8380149 × 10^−5^ × Reaction temp. − 0.0284772 × Solid content − 7.8491088 × 10^−3^ × H_2_O_2_ concent. − 4.5014496 × 10^−4^ × Reaction time + 3.4923132 × 10^−5^ × Reaction temp. × Solid content + 2.3900179 × 10^−4^ × Reaction temp. × H_2_O_2_ concent. − 2.2097257 × 10^−7^ × Reaction temp. × Reaction time + 8.6433922 × 10^−4^ × Solid content × H_2_O_2_ concent. − 4.3403413 × 10^−5^ × Solid content × Reaction time − 3.95154005 × 10^−5^ × H_2_O_2_ concent. × Reaction time − 6.01390484 × 10^−6^ × Reaction temp.^2^ + 2.664218431 × 10^−3^ × Solid content^2^ − 1.7142995 × 10^−3^ × H_2_O_2_ concent.^2^ + 2.74301920 × 10^−5^ × Reaction time^2^			
**BMP Model**			
Quadratic model			
Prob > F	<0.0001 Significant	Adj-*R*^2^	0.4112
*R*^2^	0.5728	Pred-*R*^2^	0.1190
Adeq Precision	7.23	C.V%	10.35
1/(BMP) = +4.20476 × 10^−3^ − 1.31145 × 10^−5^ × Reaction temp. − 4.36888 × 10^−5^ × Solid content − 9.28724 × 10^−4^ × H_2_O_2_ concent. − 4.17111 × 10^−5^ × Reaction time + 4.08924 × 10^−^^7^ × Reaction temp. × Solid content + 3.96470× 10^−^^6^ × Reaction temp. × H_2_O_2_ concent. − 8.50445 × 10^−^^8^ × Reaction temp. × Reaction time − 3.64937 × 10^−5^ × Solid content × H_2_O_2_ concent. − 2.27112 × 10^−^^6^ × Solid content × Reaction time + 4.32086 × 10^−^^6^ × H_2_O_2_ concent. × Reaction time + 1.02675 × 10^−^^7^ × Reaction temp.^2^ + 1.19229 × 10^−5^ × Solid content^2^ + 2.09008 × 10^−^^4^ × H_2_O_2_ concent.^2^ + 1.83088 × 10^−^^6^ × Reaction time^2^			

**Table 3:** Comparison of Fourier-transform infrared (FTIR) spectra of waste pretreated with alkaline H_2_O_2_ under different conditions with with that of raw greenhouse crop waste.

Wavelength (cm^−1^)	Region	50 °C, 5% VS, 15 h, 2% H_2_O_2_	50 °C, 7% VS, 6 h, 1% H_2_O_2_	100 °C, 3% VS, 24 h, 3% H_2_O_2_
895–900	Characteristic absorption peak of cellulose associated with the ß-glycosidic bond [19,20]	+++++	+	++++
1050	C–O stretch of the C–O–C in cellulose, hemicellulose, and lignin [19,21]	+++++	+	++
1270	C–O stretch in the guaiacyl aromatic ring associated with lignin [19,37]	+++	++	+++++
1430–1460	Aromatic skeletal vibration combined with C–H in plane deformation associated with lignin [37,38]	++++	+++	+++++
1510–1600	Aromatic skeletal vibration of lignin constituting conjugated C=C, aryl-substituted C=C, and alkenyl C=C stretch [37,38,39]	+++++	+	+++
2920–2925	C–H vibration of CH_2_ and CH_3_ groups [19,37]	+++	++	+++++
3420	Inter- and intramolecular hydrogen bonding [19]	++++	+++	+++++
3446	O–H stretch vibration in cellulose [37]	+++	+	++++

+++++ to +: Max to Min.
